# Performance of Transvaginal First-Trimester Anatomical Screening by Operators with Different Levels of Experience: A Randomized Controlled Trial

**DOI:** 10.3390/diagnostics16172769

**Published:** 2026-08-28

**Authors:** Panithi Leesukon, Wirada Dulyaphat, Woraluk Moradokkasem, Waranyu Lertrat, Sanpon Diawtipsukon, Nareenun Chansriniyom, Theera Tongsong, Chayada Tangshewinsirikul

**Affiliations:** 1 Division of Maternal-Fetal Medicine, Department of Obstetrics & Gynaecology, Faculty of Medicine Ramathibodi Hospital, Mahidol University, Bangkok 10400, Thailand; panithi.lee@mahidol.ac.th (P.L.); wirada.dul@mahidol.ac.th (W.D.); woraluk.mor@mahidol.ac.th (W.M.); waranyu.ler@mahidol.ac.th (W.L.); sanpon.dia@mahidol.ac.th (S.D.); nareenun.cha@mahidol.ac.th (N.C.); 2 Department of Obstetrics and Gynecology, Faculty of Medicine, Chiang Mai University, Chiang Mai 50200, Thailand; theera.t@cmu.ac.th

**Keywords:** first-trimester anatomical screening, transvaginal ultrasound, feasibility, learning curve, ISUOG protocol

## Abstract

**Background/Objectives:** First-trimester anatomical screening (FTAS) enables early detection of fetal structural anomalies. However, data on transvaginal ultrasound performance across different operator experience levels remains limited. The primary objective was to compare the performance of transvaginal FTAS between inexperienced and experienced operators by comparing complete scan rates and anatomical visualization scores. The secondary objectives were to compare scan duration and learning curves between the two operators and to identify factors associated with successful examinations. **Methods:** In this randomized controlled trial, participants underwent transvaginal FTAS performed either by a first-year maternal–fetal medicine (MFM) fellow (inexperienced operator) or an MFM specialist (experienced operator) according to International Society of Ultrasound in Obstetrics and Gynecology (ISUOG) guidelines. Complete scan rates, anatomical visualization scores, scan duration, factors affecting visualization, and learning curves were compared between groups. **Results:** A total of 139 participants were included in the analysis, with 70 and 69 examinations performed by the inexperienced and experienced operators, respectively. Complete scan rates and visualization scores were not significantly different between groups (41.4% vs. 44.9% and 86.4% vs. 91.2%, respectively). On multivariate analysis, operator experience was independently associated with higher visualization scores (*p* = 0.010), but not complete scan rates. Scan duration was similar between groups (median (IQR): 20 (17) vs. 20 (15) min; *p* = 0.211). Fetal presentation and uterine position significantly influenced visualization outcomes. The inexperienced operator demonstrated a trend of gradual improvement over time, though not significantly, whereas the experienced operator maintained stable performance throughout the study period. **Conclusions:** After training, no statistically significant differences were observed between less experienced and experienced operators with respect to complete scan rate, scan duration, and learning curve, although visualization score was significantly higher in the experienced examiner on multivariate analysis. However, our findings should be interpreted with caution, as the conclusions were based on the performance of only one examiner in each group.

## 1. Introduction

The traditional roles of early pregnancy ultrasound have been to establish accurate gestational age, determine chorionicity in multiple pregnancies, and measure nuchal translucency (NT) for aneuploidy screening. However, advances in transducer technology, improved image resolution, and a greater understanding of fetal anatomical development have greatly expanded the role of first-trimester ultrasound. When performed between 11 and 13^+6^ weeks of gestation, first-trimester anatomical screening (FTAS) now provides an important opportunity for the detection of major fetal structural anomalies early in pregnancy [1,2,3,4]. These scans have important clinical value. They provide early reassurance for parents when the fetus appears structurally normal. When abnormalities are suspected, they also enable timely counseling and facilitate early genetic diagnostic testing. Furthermore, in situations where pregnancy termination is considered, early assessment may reduce maternal morbidity by enabling the performance of a termination at an earlier gestational age.

To improve the detection rate, guidelines have recently been published by international professional societies, including the International Society of Ultrasound in Obstetrics and Gynecology (ISUOG) and the American Institute of Ultrasound in Medicine (AIUM) [5,6,7,8]. When performed according to guideline protocols, the combination of transabdominal and transvaginal ultrasound facilitates FTAS, with reported detection rates approaching 80% depending on the specific organ assessed [1,4,7,9,10,11].

Transvaginal ultrasound enables better visualization of fetal organs during early gestation, especially when the transabdominal examination is limited or inconclusive [5,6,12,13,14]. However, the technique can be technically challenging because of the limited maneuverability of the transvaginal probe. Previous studies using transvaginal ultrasound have shown that several factors, including gestational age and fetal position, influence the feasibility of completing a comprehensive first-trimester fetal anatomical assessment [12,15]. Previous studies confirmed the feasibility of using the transabdominal approach for FTAS and demonstrated a short learning curve for inexperienced operators [16,17]. However, studies evaluating transvaginal ultrasound, which is technically different from transabdominal ultrasound, among operators with varying levels of experience remain scarce.

The primary objective of this study was to compare the rate of complete transvaginal FTAS at 11 to 13^+6^ weeks of gestation and anatomical visualization scores between an inexperienced and an experienced operator. The secondary objectives were to compare scan duration and learning curves between the two operators and to identify factors associated with successful examinations.

## 2. Materials and Methods

**Study design:** This randomized controlled trial was conducted at the antenatal care clinic in the Department of Obstetrics and Gynaecology, Faculty of Medicine, Ramathibodi Hospital, Mahidol University, Bangkok, Thailand between August 2025 and February 2026. The study was approved by the Ramathibodi Hospital Institutional Review Board ethics committee (COA, MURA2025/221, date of approval: 10 March 2025) and registered at the Thai Clinical Trials Registry, number 20250309014.

**Participants:** Pregnant women attending our antenatal care clinic between August 2025 and February 2026 were enrolled in the study if they met the following inclusion criteria: (1) singleton pregnancy, (2) maternal age ≥ 18 years, and (3) gestational age between 11 and 13^+6^ weeks, determined by a reliable last menstrual period and crown–rump length (CRL) measurement on ultrasound. The exclusion criteria were as follows: (1) major fetal structural anomalies detected on either the first or second-trimester ultrasound scan, (2) prenatally diagnosed chromosomal abnormalities, (3) loss to follow-up or inability to complete the second-trimester scan, and (4) refusal to participate in the study.

After eligible pregnant women were enrolled by the research physician, written informed consent was obtained from all participants.

Randomization: The randomization sequence was generated by a statistician using block randomization with a block size of four and was concealed in sequentially numbered, sealed opaque envelopes. A perinatal research nurse was responsible for the allocation process and assigned participants to undergo ultrasound examination by one of two operators: an inexperienced operator (a first-year maternal-fetal medicine [MFM] fellow who, although inexperienced in first-trimester anatomical screening, had two years of experience in second-trimester anatomical screening as a general obstetrician), and an experienced operator (a certified MFM specialist with 10 years of experience in second-trimester anatomical screening and nuchal translucency measurement who had previously achieved competency in transabdominal first-trimester anatomical screening) [16]. Both operators were certified by the Fetal Medicine Foundation for 11- to 13^+6^-week scans and nuchal translucency measurement and had received standardized training in first-trimester transvaginal anatomical ultrasound according to the ISUOG guidelines [5]. Both operators were blinded to the randomization sequence.

**Intervention:** The operator performed TAS for evaluation of the general pregnancy status including fetal number, viability, crown-rump length (CRL), gestational age at the time of the scan, fetal presentation, placental location, uterine position, and the presence of uterine fibroid. Subsequently, a first-trimester fetal anatomical assessment was conducted transvaginally using a high-resolution two-dimensional 4–9 MHz convex endocavity transducer connected to a Voluson E10 ultrasound system (GE Healthcare, Zipf, Austria) with a first-trimester preset. All examinations followed the protocol outlined in the ISUOG guidelines [5]. The duration of each scan was limited to a maximum of 20 min. Both examiners recorded checklist images for all successfully visualized structures during the examination as evidence of visualization, including the NT image when an optimal plane for measurement was successfully obtained.

**Outcome measures:** Demographic and clinical data, including maternal age, nulliparity, maternal body mass index (BMI), and abdominal surgical scar, were recorded in the study record form. The visibility of each organ or anatomical structure was recorded immediately after the procedure using the checklist form. Each structure was assigned a score of 1 if it was clearly visualized and a score of 0 if it was not visualized or if the recorded image was inconclusive. For nuchal translucency measurement, a score of 1 was assigned when the structure was clearly visualized and the thickness could be measured; otherwise, a score of 0 was assigned. According to the study protocol, complete visualization of the mandatory, optional, and total fetal anatomical structures were defined as the successful assessment of 13, 27, and 40 anatomical views, respectively. The scan duration for each operator was recorded either upon completion of the full examination or after reaching the 20 min time limit. All cases in which fetal anomalies were detected during the first-trimester scan underwent repeat ultrasound examination by another MFM staff member on the same day. Prenatal diagnostic procedures were performed when indicated. The pregnancies with normal first-trimester anatomical findings that continued beyond the first trimester were confirmed to be structurally normal at the routine mid-trimester anomaly scan by other certified MFM staff members.

**Sample size estimation:** Based on a previous study by Rosati et al. [12], the complete screening capability of first-trimester transvaginal fetal anatomical scanning was approximately 79%. Accordingly, the complete screening capability was assumed to be 79% by the operator with greater experience, and a 25-percentage-rate difference was considered clinically relevant, corresponding to an assumed capability of 54% among the less-experienced operator. With a two-sided α of 0.05, 80% power, and a 1:1 allocation ratio, the required sample size was 55 pregnant women per group. Allowing for a potential 25% loss or exclusion of data, the final required sample size was 74 pregnant women per group, for a total of 148 pregnant women.

**Statistical analysis:** Continuous variables were expressed as mean ± standard deviation or median (IQR) and compared using Student’s *t*-test or Mann-Whitney_U test as appropriate. Categorical variables were expressed as number (percentage) and compared using the chi-square test. Logistic regression analysis and multivariable linear regression analysis were performed to evaluate factors associated with complete scan rate and visualization score, respectively. The primary exposure variable was operator experience. Models were adjusted for clinically relevant potential confounders, including maternal age, nulliparity, body mass index (BMI), abdominal surgical scar, gestational age at examination, fetal presentation, placental location, uterine position, and the presence of uterine fibroids, based on explanatory approach. Repeated-measures analysis was performed to evaluate changes in examiner performance over time as clinical experience accumulated. For each examiner, consecutive examinations were ordered chronologically and grouped into 14 sequential sets of 10 examinations (Sets 1–14). These sequential examination sets were treated as the time variable, representing increasing cumulative experience. For each examiner, the performance score was calculated for each 10-examination set, resulting in 14 repeated performance measurements per examiner across successive time points. Thus, the repeated observations represented longitudinal measurements of examiner performance. A repeated-measures model was used to account for the correlation among performance scores obtained from the same examiner across successive time points. The model included performance score as the continuous outcome variable, examination set (Sets 1–14) as the time variable, operator group (inexperienced versus experienced examiner) as the between-examiner factor, and the time × operator-group interaction to assess whether the pattern or rate of change in performance over successive examination sets differed between the two examiners.

Based on the final model, marginal prediction plots were generated to illustrate the estimated visualization scores from the first to the final examination set. A *p*-value < 0.05 was considered statistically significant. Finally, we performed a modified per-protocol analysis because the primary objective was to evaluate the effect of operator experience on visualization performance rather than diagnostic performance in fetuses with anomalies. Excluded cases were not omitted based on study outcomes but because the predefined study endpoint could not be validly assessed. Specifically, pregnancies complicated by miscarriage, severe fetal hydrops, or major fetal anomalies did not meet the protocol-defined conditions for evaluation. All analyses were performed using IBM SPSS Statistics for Windows, Version 26.0 (IBM Corp., Armonk, NY, USA).

## 3. Results

**Figure 1:**
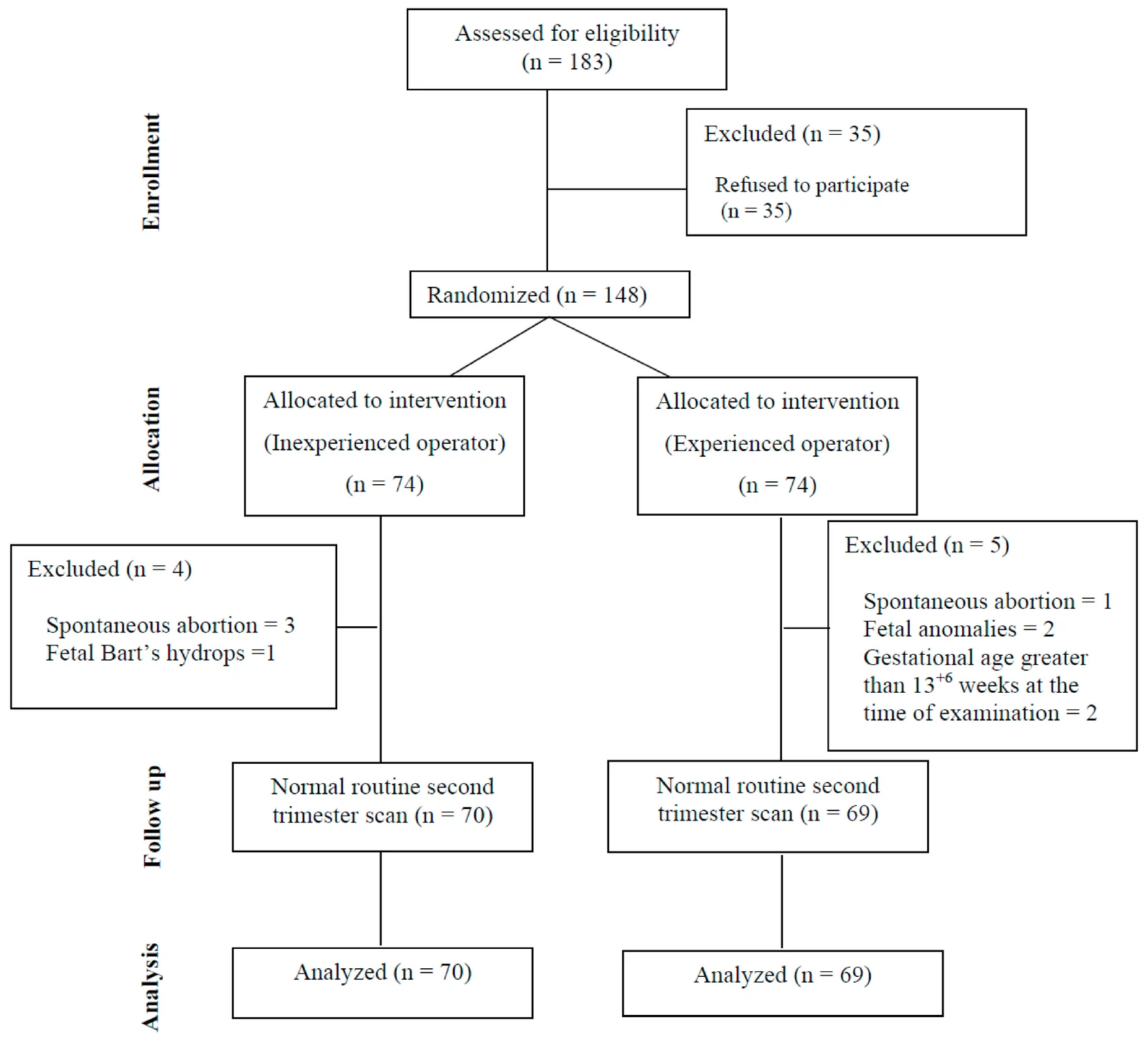
Flow chart of patient recruitment and allocation.

Among the 183 eligible pregnant women who were consecutively approached, 35 declined to participate, leaving 148 participants enrolled in the study. The participants were randomized for assessment by either the inexperienced operator (*n* = 74) or the experienced operator (*n* = 74). For the inexperienced operator, four participants were excluded due to spontaneous abortion (*n* = 3) and fetal Bart’s hydrops (*n* = 1). For the experienced operator, five participants were excluded due to spontaneous abortion (*n* = 1), fetal anomalies (*n* = 2; DiGeorge syndrome with right-sided aortic and ductal arch, and bilateral club feet), and gestational age greater than 13 + 6 weeks at the time of examination (*n* = 2). The final analysis included 70 participants for the inexperienced operator and 69 participants for the experienced operator. The CONSORT flow diagram of the study is presented in Figure 1.

Maternal and fetal baseline characteristics, including maternal age, nulliparity, BMI, abdominal surgical scar, uterine position, presence of uterine fibroids, gestational age at the time of scan, crown–rump length, and fetal presentation, were not significantly different between the two operator groups (all *p* > 0.05). However, anterior placental location was significantly more common in the inexperienced operator group than in the experienced operator group (70.0% vs. 47.8%; *p* = 0.008) (Table 1).

The rate of complete first-trimester fetal anatomy scans was not significantly different between the inexperienced operator and experienced operator (41.4% vs. 44.9%; *p* = 0.677). No significant differences were detected in the mean percentage anatomical visualization score (86.4 ± 19.3 vs. 91.2 ± 11.9; *p* = 0.078) or scan duration; median (IQR): 20 (17) vs. 20 (15) min; *p* = 0.211 (Table 2). Notably, 83 of the 139 examinations (59.7%) reached the maximum time limit of 20 min, with nearly all cases failing to achieve the optimal plane for NT measurement within the allocated time.

Visualization success rates for each fetal anatomical structure were not significantly different between the two operators, except for the thalami (inexperienced operator, 78.6% vs. experienced operator, 91.3%; *p* = 0.036), forehead (72.9% vs. 94.2%; *p* = 0.001), and upper lip (67.1% vs. 87.0%; *p* = 0.006), which were visualized more frequently by the experienced operator. Both operators were able to visualize most fetal structures, with visualization rates ranging from 70% to 100% for each structure, except for nuchal translucency (NT), which was visualized in approximately 60% of the cases (Table 3).

Both the univariate and multivariate analyses revealed no significant association between operator experience and the rate of complete scans (Table 4). However, operator experience was associated with a trend toward improved anatomical visualization scores on univariate analysis (Table 5) and was significantly associated with higher visualization scores on multivariate analysis, with an adjusted coefficient of 2.609 (*p* = 0.010) (Table 5).

Notably, fetal presentation and uterine position significantly influenced the completeness of FTAS according to both the univariate and multivariate analyses (Table 4). Fetal transverse presentation was associated with a higher likelihood of successful examination, with an adjusted odds ratio of 1.70, whereas a retroverted uterus was associated with a slight reduction in the success rate (0.82). Similarly, when the anatomical visualization score was analyzed as the outcome, fetal transverse lie was significantly associated with higher scores, whereas a retroverted uterus was significantly associated with lower scores in both univariate and multivariate analyses (Table 5). Nevertheless, these findings should be interpreted with caution due to the wide confidence intervals, which likely reflect the limited sample size.

**Figure 2:**
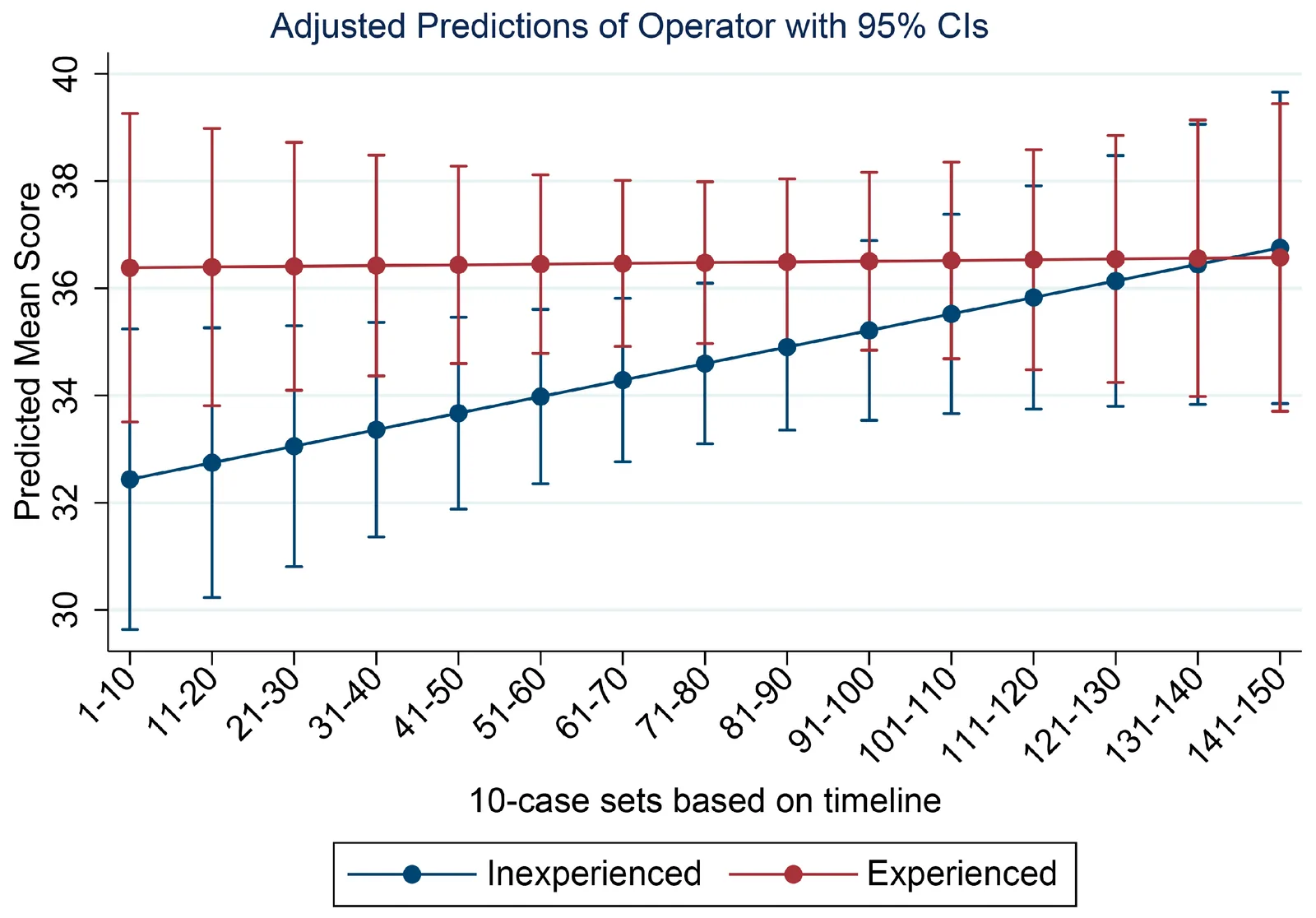
Comparison of learning curve between the inexperienced and experienced operator based on the predictive model of visualization scores.

Learning curve analysis showed a trend toward improvement of visualization scores based on the parsimonious predictive model of repeated measurement (Table 6). Figure 2 illustrates the learning curves of each operator, expressed as the predicted mean anatomical visualization scores across sequential sets of 10 examinations. For the inexperienced operator, the predicted mean score demonstrated a progressive increase over time, from an initially lower level compared with the experienced operator with a trend to be the same by the end of the study, although the initial difference did not reach statistical significance (*p* = 0.061). In contrast, the learning curve of the experienced operator remained relatively stable throughout the study period. Nevertheless, the within-operator changes in the learning curves were not statistically significant for either the inexperienced operator (*p* = 0.082) or the experienced operator (*p* = 0.241).

## 4. Discussion

Although the rates of complete FTAS were not significantly different between the two operators, the experienced operator showed a consistent trend toward higher complete scan rates, visualization scores, and superior performance during the initial phase of the learning curve. Although operator experience was not statistically significant in the univariate analysis, it remained independently associated with higher visualization scores after adjustment for potential confounding factors in the multivariable analysis. These findings suggest that prior experience has a modest yet statistically significant influence on examination performance following standardized training. Nevertheless, the magnitude of this effect appears limited and is unlikely to be clinically significant. Notably, both operators were able to visualize more than 80% of the fetal anatomical structures according to the ISUOG guidelines within a time limit of 20 min. Although the rates of complete visualization were relatively low (40–45%), both operators had acceptable scores for the mean percentage visualization [16,17,18]. The findings confirmed that FTAS is feasible for obstetricians following standardized training, with acceptable performance achieved even during the early phase of the learning curve.

Both operators were able to visualize almost all mandatory (90%) and optional (70–100%) anatomical structures recommended in the guidelines. Consequently, most fetal anomalies that are detectable in the first trimester can possibly be identified using the transvaginal approach [1,3,4]. However, NT, as one of the mandatory anatomical structures, was successfully visualized and measured in only 58% of cases. This relatively low visualization rate may be attributable to the stringent requirements for NT assessment [5]. Unlike other anatomical structures, NT measurement requires an exact mid-sagittal plane, which often necessitates additional examination time, particularly when the fetus is in an unfavorable position. In contrast, the remaining structures required only visualization without precise measurement. In routine clinical practice, obtaining an optimal NT measurement often requires additional time to allow the patient to rest, to encourage changes in activity, or to schedule a repeat examination. These findings suggest that, despite acceptable overall anatomical visualization following standardized training, a transvaginal FTAS alone may not be sufficient to achieve a complete examination, including NT evaluation, in all patients. Therefore, transvaginal FTAS should be considered a complementary imaging approach rather than a stand-alone examination and may need to be combined with transabdominal scanning or repeat assessment when complete visualization cannot be achieved.

The learning curve analysis demonstrated consistently stable scores for the experienced operator throughout the study period. In contrast, the inexperienced operator showed progressive improvement, with scores initially lower than those of the experienced operator with a trend to improve by the end of the study. Nevertheless, despite the observance of improved performance with increasing practice, the score differences across the study timeline were not statistically significant, even during the initial phase of training. These findings suggest that general obstetricians with prior experience in second-trimester anomaly screening appeared to demonstrate a short learning curve for FTAS and achieved performance levels to those of experienced operators when standardized guidelines are implemented.

A notable finding was that the learning curve of the experienced operator remained stable throughout the study period. This implies that successful anatomical visualization may be limited by several factors, particularly fetal presentation and uterine position, that cannot be modified during the examination, as suggested in previous studies [12]. Although transvaginal ultrasound generally provides superior image resolution compared with transabdominal ultrasound, our recommendation is that both approaches should be used complementarily to maximize anatomical visualization and enhance the opportunity for early detection of fetal anomalies [5,6,12,13,14]. Furthermore, additional examination time may be required in cases with suspected abnormalities to allow for more comprehensive evaluation and confirmation of the findings.

This study has several strengths. To the best of our knowledge, it is among the first studies to evaluate transvaginal ultrasound for FTAS performed by operators with different levels of experience while following the comprehensive ISUOG checklist protocol. The randomized controlled trial design and comparable baseline maternal and fetal characteristics between the two groups helped to reduce potential confounding factors. In addition, both operators underwent standardized training before study initiation, which may have reduced the variability related to the examination technique. Furthermore, the use of a structured 40-item checklist based on the ISUOG guidelines provided a standardized framework for fetal anatomical assessment.

The study also had several limitations. First, the inclusion of only two operators may limit the generalizability of the results. Second, the sample size was relatively small for multivariable analysis, particularly given the large number of potential confounding variables requiring adjustment. Third, interobserver variability in the same participants was not analyzed because of safety and ethical concerns related to prolonged transvaginal ultrasound examination during the first trimester. However, the random allocation of the participants to each study group and the comparable baseline maternal and fetal characteristics between the groups helped minimize potential selection bias and confounding factors.

From a clinical and educational perspective, this study supports the feasibility of FTAS and suggests that it can be incorporated into MFM training programs as either an optional or a routine component of screening practice. From a research perspective, the favorable findings of the present study should be interpreted in light of the aforementioned limitations. Further studies are warranted involving larger sample sizes and a greater number of operators with varying levels of experience to clarify the inconsistent findings and further validate the results. Finally, a dedicated equivalence or non-inferiority study is required to formally demonstrate comparable performance between operators with different levels of experience. Additionally, future research should focus on developing and validating objective quality-assurance tools to further improve the standardization and reproducibility of first-trimester transvaginal fetal anatomical ultrasound. Emerging artificial intelligence (AI)-based technologies, including AI-assisted plane identification and automated fetal anatomical measurements, have shown promise in reducing operator-dependent variability. However, further validation is required to establish their accuracy, reliability, and clinical applicability before routine implementation. Importantly, performance varies across anatomical structures. For example, SonoCNS™ demonstrates good reproducibility for fetal skull biometry (BPD, HC, and OFD) but lower accuracy for intracranial measurements, such as ventricular width, transcerebellar diameter, and cisterna magna [19]. Similarly, the reproducibility of Fetal HQ varies depending on the specific parameters assessed and may be influenced by operator experience and technical factors [20]. Overall, despite rapid advances, AI-based approaches remain insufficiently validated, particularly for routine fetal ultrasound screening.

## 5. Conclusions

Operator experience had a modest yet statistically significant impact on the performance of first-trimester anatomical scans. No statistically significant differences were observed between less experienced and experienced operators with respect to complete scan rate. Both operators achieved acceptable complete scan rates and anatomical visualization scores of approximately 80%, and similar scan durations. Fetal presentation and uterine version were significant factors associated with scan performance. The results of this study suggest that, in selected operators with previous second-trimester anomaly scan experience and FMF certification, standardized training may enable acceptable mean visualization scores for transvaginal FTAS according to the ISUOG guidelines within a 20-min examination. However, transvaginal FTAS alone did not achieve a high completion rate for the full examination protocol, including NT assessment, and independent validation is required.

## Figures and Tables

**Table 1:** Maternal and fetal baseline characteristics.

Baseline Characteristics	Inexperienced Operator (*N* = 70)	Experienced Operator (*N* = 69)	*p*-Value
Maternal age (years), mean ± SD	31.9 ± 4.9	32.3 ± 5.1	0.683
Nulliparous, *n* (%)	42 (60.0)	41 (59.4)	0.285
Maternal BMI (kg/m^2^), mean ± SD	25.9 + 6.1	24.6 + 4.7	0.170
Abdominal surgical scar, *n* (%)			0.451
• Pfannenstiel, *n* (%)	10 (14.3)	15 (21.7)	
• Low midline, *n* (%)	3 (4.3)	4 (5.8)	
CRL (mm), mean ± SD	66.6 ± 9.1	68.3 ± 7.9	0.239
GA at first trimester scan (days), mean ± SD	89.7 ± 4.3	90.9 ± 3.6	0.074
Presentation			0.267
• Cephalic, *n* (%)	10 (14.3)	16 (23.2)	
• Breech, *n* (%)	21 (30.0)	23 (33.3)	
• Transverse, *n* (%)	39 (55.7)	30 (43.5)	
Placenta location			0.008
• Anterior, *n* (%)	49 (70)	33 (47.8)	
• Posterior, *n* (%)	21 (30)	36 (52.2)	
Uterine position			0.138
• Anteversion, *n* (%)	66 (94.3)	60 (87.0)	
• Retroversion, *n* (%)	4 (5.7)	9 (13.0)	
Present of uterine fibroid, *n* (%)	4 (5.7)	6 (8.7)	0.361

BMI, body mass index; CRL, crown-rump length; GA, gestational age.

**Table 2:** Complete scan, score, time between two operators with different experience in routine second trimester anatomy scan.

	Inexperienced Operator (*N* = 70)	Experienced Operator (*N* = 69)	*p*-Value
Complete scan, *n* (%)	29 (41.4)	31 (44.9)	0.677
% Scores, mean ± SD	86.36 ± 19.29	91.20 ± 11.85	0.078
Time (min), median (IQR)	20 (17)	20 (15)	0.211

**Table 3:** Visualization success rate of fetal organ/anatomical area or structures between the two operators.

	Total (%)	Inexperienced Operator (*N* = 70)	Experienced Operator (*N* = 69)	*p*-Value
Confirm singleton pregnancy *	139 (100.0)	70 (100.0)	69 (100.0)	-
Overview of fetus, uterus and placenta	139 (100.0)	70 (100.0)	69 (100.0)	-
Calcification of cranium *	136 (97.8)	67 (95.7)	69 (100.0)	0.082
Contour/shape of cranium *	135 (97.1)	67 (95.7)	68 (98.6)	0.317
Interhemispheric falx *	133 (95.7)	65 (92.9)	68 (98.6)	0.099
Choroid plexus *	133 (95.7)	65 (92.9)	68 (98.6)	0.099
Thalami	118 (84.9)	55 (78.6)	63 (91.3)	0.036
Brainstem	101 (72.7)	49 (70.0)	52 (75.4)	0.478
Cerebral peduncle with aqueduct of Sylvius	118 (84.9)	56 (80.0)	62 (89.9)	0.105
Intracranial translucency	120 (86.3)	57 (81.4)	63 (91.3)	0.090
Cisterna magna	119 (85.6)	56 (80.0)	63 (91.3)	0.058
Forehead	116 (83.5)	51 (72.9)	65 (94.2)	0.001
Bilateral orbits	134 (96.4)	66 (94.3)	68 (98.6)	0.177
Nasal bone	107 (77.0)	50 (71.4)	57 (82.6)	0.117
Maxilla	120 (86.3)	62 (88.6)	58 (84.1)	0.439
Retronasal triangle	128 (92.1)	66 (94.3)	62 (89.9)	0.333
Upper lip	107 (77.0)	47 (67.1)	60 (87.0)	0.006
Mandible	113 (81.3)	55 (78.6)	58 (84.1)	0.407
Nuchal translucency *	81 (58.3)	41 (58.6)	40 (58.0)	0.943
No jugular cysts	122 (87.8)	59 (84.3)	63 (91.3)	0.207
Shape of thoracic wall	123 (88.5)	61 (87.1)	62 (89.9)	0.616
Lung fields	122 (87.8)	60 (85.7)	62 (89.9)	0.456
Diaphragm	133 (95.7)	68 (97.1)	65 (94.2)	0.394
Heart activity *	138 (99.3)	69 (98.6)	69 (100.0)	0.319
Situs	121 (87.1)	59 (84.3)	62 (89.9)	0.328
Position with axis	119 (85.6)	58 (82.9)	61 (88.4)	0.351
Size	120 (86.3)	58 (82.9)	62 (89.9)	0.230
Four-chamber view *	121 (87.1)	58 (82.9)	63 (91.3)	0.138
Left ventricular outflow tract	100 (71.9)	46 (65.7)	54 (78.3)	0.100
Three-vessel-and-trachea view	113 (81.3)	54 (77.1)	59 (85.5)	0.206
No tricuspid regurgitation	108 (77.7)	51 (72.9)	57 (82.6)	0.167
Positive a-wave of DV	134 (96.4)	67 (95.7)	67 (97.1)	0.661
Stomach *	133 (95.7)	65 (92.9)	68 (98.6)	0.099
Bladder *	126 (90.6)	65 (92.9)	61 (88.4)	0.367
Intact abdominal wall *	137 (98.6)	68 (97.1)	69 (100.0)	0.157
Two umbilical arteries	126 (90.6)	65 (92.9)	61 (88.4)	0.367
Bilateral kidneys	129 (92.8)	65 (92.9)	64 (92.8)	0.981
Spine	135 (97.1)	67 (95.7)	68 (98.6)	0.317
Upper limb *	139 (100.0)	70 (100.0)	69 (100.0)	-
Lower limbs *	139 (100.0)	70 (100.0)	69 (100.0)	-
Success: Total (40 items)	60 (43.2)	29 (41.4)	31 (44.9)	0.677
Success: Mandatory (13 items)	75 (54.0)	38 (54.3)	37 (53.6)	0.938

“*” indicates a mandatory item.

**Table 4:** Factors influencing complete first trimester fetal anatomy scan.

	Univariable Analysis			Multivariable Analysis		
Factors	Odd Ratio	95%CI	*p*-Value	a Odd Ratio	95%CI	*p*-Value
Operator experience	1.04	0.88; 1.22	0.679	1.07	0.092; 1.24	0.355
Maternal age	0.99	0.97; 1.00	0.129	-	-	-
Nulliparous	1.04	0.88; 1.23	0.639	-	-	-
Maternal BMI	0.99	0.97; 1.00	0.075	0.99	0.97; 1.02	0.170
Abdominal scar (Control = No scar)						
• Pfannenstiel	1.07	0.86; 1.33	0.535	-	-	-
• Low midline	1.17	0.80; 1.72	0.410	-	-	-
GA at first trimester scan	0.99	0.97; 1.01	0.390	1.00	0.98; 1.02	0.960
Presentation (Control = Vertex)						
• Breech	1.08	0.87; 1.33	0.501	1.15	0.95; 1.38	0.150
• Transverse	1.67	1.37; 2.04	<0.001	1.70	1.42; 2.03	<0.001
Placental location (Control = anterior)						
• Posterior	1.14	0.96; 1.35	0.125	1.14	0.98; 1.34	0.092
Uterine position (Control = anteversion)						
• Retrovert	0.74	0.56; 0.97	0.032	0.82	0.69; 0.98	0.028
Uterine fibroid (Control = No)						
• Anterior wall	0.85	0.58; 1.24	0.395	0.85	0.62; 1.16	0.306
• Posterior wall	0.64	0.24; 1.70	0.368	0.77	0.58; 1.02	0.066
• Anterior and posterior wall	0.64	0.32; 1.28	0.204	0.95	0.81; 1.12	0.530

BMI, body mass index; GA, gestational age.

**Table 5:** Factors influencing scores of first trimester fetal anatomy scan.

	Univariable Analysis			Multivariable Analysis		
Factors	Coefficient	95%CI	*p*-Value	a Coefficient	95%CI	*p*-Value
Operator experience	1.935	−0.197; 4.068	0.075	2.609	0.619; 4.600	0.010
Maternal age	−0.229	−0.443; 0.015	0.056	-	-	-
Nulliparous	−0.064	−2.228; 2.099	0.953	-	-	-
Maternal BMI	−0.199	−0.396; −0.003	0.046	−0.146	−0.329; 0.0358	0.115
Abdominal scar (Control = No scar)						
• Pfannenstiel	−1.365	−4.182; 1.452	0.342	-	-	-
• Low midline	2.069	−2.878; 7.017	0.412	-	-	-
GA at first trimester scan	−0.018	−0.287; 0.252	0.898	0.502	−0.163; 0.332	0.502
Presentation (Control = Vertex)						
• Breech	1.135	−1.727; 3.996	0.437	1.564	−1.266; 4.395	0.279
• Transverse	6.102	3.440; 8.764	<0.001	6.217	3.608; 8.825	<0.001
Placental location (Control = anterior)						
• Posterior	1.169	−1.015; 3.353	0.294	0.709	−1.305; 2.722	0.490
Uterine position (Control = anteversion)						
• Retrovert	−6.156	−9.714; −0.598	0.001	−5.139	−8.471; −1.808	0.003
Uterine fibroid (Control = No)						
• Anterior wall	−0.477	−5.146; 4.462	0.850	−0.136	−4.508; 4.237	0.951
• Posterior wall	−0.620	−13.396; 12.155	0.924	0.810	−10.734; 12.355	0.891
• Anterior and posterior wall	−6.120	−15.189; 2.948	0.186	−1.650	−9.858; 6.559	0.694

BMI, body mass index; GA, gestational age.

**Table 6:** Predictive model for visualization scores, using experienced operator as the reference base for comparison.

Scores	Coef.	Std. Err.	z	*p*-Value	95% Conf. Interval
Inexperienced operator	−4.240	2.267	−1.87	0.061	−8.683 to 0.202
Set number of practices	0.014	0.178	0.08	0.939	−0.336 to 0.363
Inexperienced operator × Set number of practices	0.295	0.251	1.17	0.241	−0.198 to 0.787
Constant	36.369	1.623	22.41	0.000	33.189 to 39.549

## Data Availability

The datasets generated and/or analyzed during the current study are available from the corresponding author upon direct request.

## References

[1] Syngelaki A., Hammami A., Bower S., Zidere V., Akolekar R., Nicolaides K.H. (2019). Diagnosis of fetal non-chromosomal abnormalities on routine ultrasound examination at 11–13 weeks’ gestation. Ultrasound Obstet. Gynecol..

[2] Pilalis A., Basagiannis C., Eleftheriades M., Faros E., Troukis E., Armelidou E., Papastefanou I., Souka A.P. (2012). Evaluation of a two-step ultrasound examination protocol for the detection of major fetal structural defects. J. Matern. Fetal Neonatal Med..

[3] Syngelaki A., Chelemen T., Dagklis T., Allan L., Nicolaides K.H. (2011). Challenges in the diagnosis of fetal non-chromosomal abnormalities at 11–13 weeks. Prenat. Diagn..

[4] Karim J.N., Di Mascio D., Roberts N., Papageorghiou A.T. (2024). Detection of non-cardiac fetal abnormalities on ultrasound at 11–14 weeks: Systematic review and meta-analysis. Ultrasound Obstet. Gynecol..

[5] Bilardo C.M., Chaoui R., Hyett J.A., Kagan K.O., Karim J.N., Papageorghiou A.T., Poon L.C., Salomon L.J., Syngelaki A., International Society of Ultrasound in Obstetrics and Gynecology (2023). ISUOG Practice Guidelines (updated): Performance of 11–14-week ultrasound scan. Ultrasound Obstet. Gynecol..

[6] (2021). AIUM Practice Parameter for the Performance of Detailed Diagnostic Obstetric Ultrasound Examinations Between 12 Weeks 0 Days and 13 Weeks 6 Days. J. Ultrasound Med..

[7] Karim J.N., Roberts N.W., Salomon L.J., Papageorghiou A.T. (2017). Systematic review of first-trimester ultrasound screening for detection of fetal structural anomalies and factors that affect screening performance. Ultrasound Obstet. Gynecol..

[8] Salomon L.J., Alfirevic Z., Bilardo C.M., Chalouhi G.E., Ghi T., Kagan K.O., Lau T.K., Papageorghiou A.T., Raine-Fenning N.J., Stirnemann J. (2013). ISUOG practice guidelines: Performance of first-trimester fetal ultrasound scan. Ultrasound Obstet. Gynecol..

[9] Petousis S., Sotiriadis A., Margioula-Siarkou C., Tsakiridis I., Christidis P., Kyriakakis M., Mamopoulos A., Athanasiadis A., Dagklis T. (2020). Detection of structural abnormalities in fetuses with normal karyotype at 11–13 weeks using the anatomic examination protocol of the International Society of Ultrasound in Obstetrics and Gynecology (ISUOG). J. Matern. Fetal Neonatal Med..

[10] Chen F.C., Bacovsky A., Entezami M., Henrich W. (2019). Nearly half of all severe fetal anomalies can be detected by first-trimester screening in experts’ hands. J. Perinat. Med..

[11] Kenkhuis M.J.A., Bakker M., Bardi F., Fontanella F., Bakker M.K., Fleurke-Rozema J.H., Bilardo C.M. (2018). Effectiveness of 12–13-week scan for early diagnosis of fetal congenital anomalies in the cell-free DNA era. Ultrasound Obstet. Gynecol..

[12] Rosati P., Guariglia L., Bertuzzi A. (2000). Transvaginal assessment of fetal anatomy at 11 to 16 weeks of gestation in relation to fetal position. Fetal Diagn. Ther..

[13] García Fernández S., Arenas Ramirez J., Otero Chouza M.T., Rodriguez-Vijande Alonso B., Llaneza Coto Á.P. (2019). Early fetal ultrasound screening for major congenital heart defects without Doppler. Eur. J. Obstet. Gynecol. Reprod. Biol..

[14] Buskmiller C., Fishel Bartal M., Bonilla M., Denham C., Nguyen R., Sibai B., Pedroza C., Hernandez-Andrade E. (2023). First trimester anatomy ultrasound for patients with obesity: A randomized controlled trial. Am. J. Obstet. Gynecol. MFM.

[15] McAuliffe F.M., Fong K.W., Toi A., Chitayat D., Keating S., Johnson J.A. (2005). Ultrasound detection of fetal anomalies in conjunction with first-trimester nuchal translucency screening: A feasibility study. Am. J. Obstet. Gynecol..

[16] Sripilaipong S., Panburana P., Wattanayingcharoenchai R., Tangshewinsirikul C. (2022). Feasibility and learning curve of performing first trimester fetal anatomy screening among operators with varying experience using the protocol of the International Society of Ultrasound in Obstetrics and Gynecology (ISUOG). J. Matern. Fetal Neonatal Med..

[17] Abu-Rustum R.S., Ziade M.F., Abu-Rustum S.E. (2011). Learning curve and factors influencing the feasibility of performing fetal echocardiography at the time of the first-trimester scan. J. Ultrasound Med..

[18] Everwijn S.M., van Bohemen J.F., Jansen F.A., Steggerda S.J., Teunissen A.K., Haak M.C. (2024). Feasibility of neurosonography in CHD-fetuses and controls in a clinical tertiary setting. Eur. J. Obstet. Gynecol. Reprod. Biol. X.

[19] Mlodawski J., Zmelonek-Znamirowska A., Mlodawska M., Detka K., Białek K., Swiercz G. (2024). Repeatability and reproducibility of artificial intelligence-acquired fetal brain measurements (SonoCNS) in the second and third trimesters of pregnancy. Sci. Rep..

[20] Mlodawski J., Zmelonek-Znamirowska A., Pawlik L., Mlodawska M., Swiercz G. (2025). Reproducibility Challenges in Fetal Cardiac Function Analysis with 2D Speckle-Tracking Echocardiography: Insights from FetalHQ. J. Clin. Med..

